# Multidisciplinary care pathways for falls prevention in older adults: visualizing the needs of primary care-based health care professionals

**DOI:** 10.1007/s41999-024-01142-3

**Published:** 2025-01-09

**Authors:** Sara S. Groos, Stefanie M. Tan, Annemiek J. Linn, Judith I. Kuiper, Natasja M. van Schoor, Julia C. M. van Weert, Nathalie van der Velde

**Affiliations:** 1 Internal Medicine, Section of Geriatric Medicine, Amsterdam UMC Location University of Amsterdam, Amsterdam, The Netherlands; 2 Amsterdam Public Health Research Institute, Amsterdam, The Netherlands; 3 Amsterdam School of Communication Research/ASCoR, University of Amsterdam, Amsterdam, The Netherlands; 4 VeiligheidNL, Amsterdam, The Netherlands; 5 Epidemiology and Data Science, Amsterdam UMC Location Vrije Universiteit Amsterdam, Amsterdam, The Netherlands

**Keywords:** Falls risk stratification, Multifactorial falls risk assessment, Multidisciplinary care, Falls prevention, Journey mapping

## Abstract

**Purpose:**

Multidisciplinary care pathways for falls prevention, which include falls risk stratification, multifactorial falls risk assessment, and management of multidomain interventions, can reduce falls in older adults. However, efficient multidisciplinary falls prevention care is challenging due to issues such as poor communication and role allocation. This study aimed to identify and visualize the multidisciplinary care needs of primary care-based health care professionals (HCPs) for falls prevention in the Netherlands using the novel co-design approach of journey mapping.

**Methods:**

Online focus groups and interviews (*N* = 45) were conducted with physical therapists (*n* = 15), district nurses (*n* = 9), occupational therapists (*n* = 7), pharmacists (*n* = 6), nurse practitioners (*n* = 5), podiatrists (*n* = 2), and one general practitioner. HCPs were asked about their interactions, experiences, needs, and barriers with regards to multidisciplinary falls prevention care in a primary care context. Insights were used to visualize a journey map depicting the desired future state of multidisciplinary care pathways for falls prevention.

**Results:**

Journey mapping identified the following needs for effective multidisciplinary falls prevention care: a dedicated case manager after risk stratification, preparatory patient information before the assessment, small multidisciplinary care team for the assessment, patient involvement during intervention management, good communication between HCPs, and a reduction in workload for HCPs.

**Conclusion:**

The inclusion of a case manager program for older adults and access to resources to facilitate good communication between HCPs are important to optimize the configuration of multidisciplinary care pathways for falls prevention in actual practice.

**Supplementary Information:**

The online version contains supplementary material available at 10.1007/s41999-024-01142-3.

## Key summary points

**Aim:**

The aim of this study was to gain insight into and visualize the needs of primary care-based health care professionals with regards to multidisciplinary falls prevention care using journey mapping.

**Findings:**

A case manager program, preparatory patient information, small multidisciplinary care team, patient involvement, good communication, and decreased workload are needed for efficient multidisciplinary falls prevention care.

**Message:**

The findings provide important implications for policy-makers seeking to develop sustainable multidisciplinary care pathways for falls prevention in primary care.

**Supplementary Information:**

The online version contains supplementary material available at 10.1007/s41999-024-01142-3.

## Introduction

Falls and related injuries contribute significantly to morbidity and mortality in older adults (65+) [1]. Falls are usually a result of several, modifiable risk factors, such as reduced mobility, vision, and use of medications, which makes their management and prevention complex [2]. With the global population aging, the prevalence of falls in older adults is expected to increase as the incidence rate of multimorbidity, polypharmacy, and frailty among this age group increases [3]. This makes falls in older adults a significant global health problem, and effective falls prevention interventions are vital to ensure improved health outcomes for older adults in the Netherlands and reduce the burden on the Dutch health care system [4].

Geriatric multidisciplinary care involves the collaboration between HCPs from multiple disciplines on a single case. This approach is recommended for health problems that are multifactorial in nature, such as falls, to effectively address the complex care needs associated with such conditions [5, 6]. Research trials have shown that multidisciplinary care pathways for falls prevention are more effective in reducing the rate of falls compared to single-care or care as usual [7–9]. Multidisciplinary care pathways for falls prevention include a falls risk stratification to identify high falls risk, a multifactorial falls risk assessment to detect individual risk factors, and the management of multidomain interventions to target detected falls risk factors [3, 10]. Despite its effectiveness in research trials, efficient multidisciplinary care for falls prevention in older adults in practice remains challenging. HCPs are uncertain about the distribution of roles and responsibilities that constitute multidisciplinary falls prevention care [11]. Furthermore, HCPs have been shown to experience poor communication and coordination within multidisciplinary falls prevention care [11, 12], suggesting that such falls prevention interventions have yet to be optimized for primary care-based HCPs in practice.

Research suggests that involving users in the development of interventions could enhance adoption and implementation in practice through improved intervention design [13, 14]. Journey mapping is a novel co-design approach that relies on the direct involvement of users. This method translates various components of the user journey into a visual map that can be used to inform the development and implementation of interventions [15, 16]. Journey mapping has been useful in the context of falls prevention by informing the integration of a falls risk management computerized clinical decision support system in primary care. Specifically, this previous study used journey mapping to provide a significant understanding into general practitioners (GPs) and nurse practitioners falls risk management experience, and the desired future state of the system [17]. However, the study did not explore the needs of other primary care-based HCPs commonly involved in falls risk and factors management, such as occupational therapists and district nurses. Addressing this gap is essential for effectively assisting policy-makers in developing falls prevention interventions that are acceptable to all end users (i.e., primary care-based HCPs). For this reason, the aim of this study is to identify the multidisciplinary falls prevention care needs of primary care-based HCPs by constructing a visual journey map that outlines the desired future state of multidisciplinary care pathways for falls prevention (i.e., for falls risk stratification, multifactorial falls risk assessment, and management of multidomain interventions) using a Dutch primary care context.

## Methods

### Procedure

A total of eight online focus groups and five online interviews (*N* = 45) were conducted between February 2022 and January 2023 using Zoom. Where necessary, interviews were offered as an alternative to focus groups to accommodate to the schedules of HCPs. HCPs were recruited with assistance from a total of 13 representatives from nine different HCP organizations who were asked to distribute a call for participants (see online appendix for the full list of organizations). Each organization is in direct contact with different primary care-based HCPs in all 12 provinces of the Netherlands. This call for participants included information about the study and a sign-up sheet. HCPs were eligible to participate and contacted for informed consent if they were currently (1) working in Dutch primary care; and (2) regularly engage in falls prevention care with older adults. The study was assessed by the Medical Ethics Research Committee of the Amsterdam University Medical Center (Amsterdam UMC, location University of Amsterdam; W21_179 # 21.194) who declared that the Medical Research Involving Human Subjects Act did not apply to this study. Written or verbal informed consent was obtained from all participants prior to data collection.

### The Fall Analysis

This study was conducted in the Dutch primary care setting. Based on Dutch guideline recommendations, multidisciplinary care pathways for falls prevention (i.e., for risk stratification, assessment, and intervention management) can be carried out in primary care by HCPs with experience in falls prevention, such as by GPs, nurse practitioners, physical therapists, or occupational therapists, using the Fall Analysis [18]. Developed by the Dutch National Expert Center on Injury Prevention (in Dutch: VeiligheidNL), the Fall Analysis (in Dutch: ‘Valanalyse’) is a validated-instrument that guides HCPs in identifying older adults at high risk of falling followed by the assessment of 13 (modifiable) risk factors for falls in these older adults (i.e., history of falls, mobility, medication, dizziness/vestibular, vision, hearing, cognition, urinary incontinence, fear of falling, activities of daily living, environmental factors, footwear and foot problems, nutritional status and vitamin D intake). Risk factors are assessed using a set of evidence-based questionnaires and functional assessments, such as the Short Physical Performance Battery or Short Nutritional Assessment Questionnaire 65^+^ for assessing mobility and nutritional status, respectively. Based on the multifactorial falls risk assessment, interventions are selected, which are intended to reduce falls risk by targeting identified risk factors [19].

### Focus group and interview sessions

Focus groups were held with physical therapists only (*n* = 2 focus groups), pharmacists only (*n* = 1), district nurses only (*n* = 1), nurse practitioners only (*n* = 2), and a mix of different HCPs (*n* = 2), and were carried out by two moderators (SG and LW, SG and ST). The interviews were held with occupational therapists (*n* = 4) and a GP, and were carried out by one interviewer (SG or ST). To prepare participants, slides and instructions for using Zoom were developed and shared with participants via email. The preparatory slides provided an overview of the Fall Analysis and the topics to be discussed during the focus groups/interviews. A semi-structured focus group/interview guide was developed following an iterative process whereby the session topics were based on the previous research examining the needs of Fall Analysis users [20].

The focus groups and interviews started with a short introduction on the topics to be discussed. HCPs were asked about their multidisciplinary falls prevention care needs for the Fall Analysis. The topics focused on understanding HCPs interactions, experiences, needs, and barriers for each pathway component (i.e., for falls risk stratification, multifactorial falls risk assessment, and management of multidomain interventions; see online appendix for guide). For this, participants were shown a journey map for the Fall Analysis from start (i.e., falls risk stratification) to finish (i.e., management of multidomain interventions), as displayed in Fig. S1 in the online appendix. Participants were asked to think about how they would like to conduct the Fall Analysis (e.g., where, how, and who should stratify for falls risk?). Participants in the focus group sessions were divided into separate, online groups using virtual breakout rooms in which they could discuss the questions. Afterward, one member from each group shared the groups desired future state of the Fall Analysis with the remainder of focus group participants. Subsequently, a discussion unfolded and the journey map was updated based on the discussion. Participants in the interviews engaged in co-design with the interviewer.

### Data analysis

All focus group and interview sessions were recorded and transcribed verbatim by two of the moderators (SG, ST). All transcripts were analyzed in MAXQDA 2022 [21]. Each transcript was coded by one of the two moderators (SG, ST) using a general inductive approach [22]. The coding was discussed in regular meetings (between SG and ST), and disagreements were resolved through discussion. Coding first began with a thematic analysis to examine detailed needs within the raw data using a main category (e.g., assessment location), and where necessary, sub-category coding scheme (e.g., home). The results from the thematic analysis helped to identify specific needs for each pathway component (i.e., for falls risk stratification, multifactorial falls risk assessment, and management of multidomain interventions), and the similarities and differences between HCPs within these pathway components. These insights were used to develop four preliminary journey maps illustrating the desired future state of multidisciplinary care pathways for falls prevention for different HCPs (e.g., nurse practitioners versus physical therapists). The preliminary journey maps were subsequently combined into one journey map, and further clarification was added from the co-design sessions. After several rounds of iterations, a condensed journey map was developed depicting the desired future state of multidisciplinary care pathways for falls prevention that are in line with the multidisciplinary falls prevention care needs of primary care-based HCPs in the Netherlands.

## Results

The final sample included 45 HCPs. The majority of participants were physical therapists (33%, *n* = 15) followed by district nurses (20%, *n* = 9), occupational therapists (16%, *n* = 7), pharmacists (13%, *n* = 6), nurse practitioners (11%, *n* = 5), podiatrists (4%, *n* = 2), and a GP (2%, *n* = 1). The results are discussed below using illustrative quotes that have been translated into English where applicable.

### Journey map

**Fig. 1:**
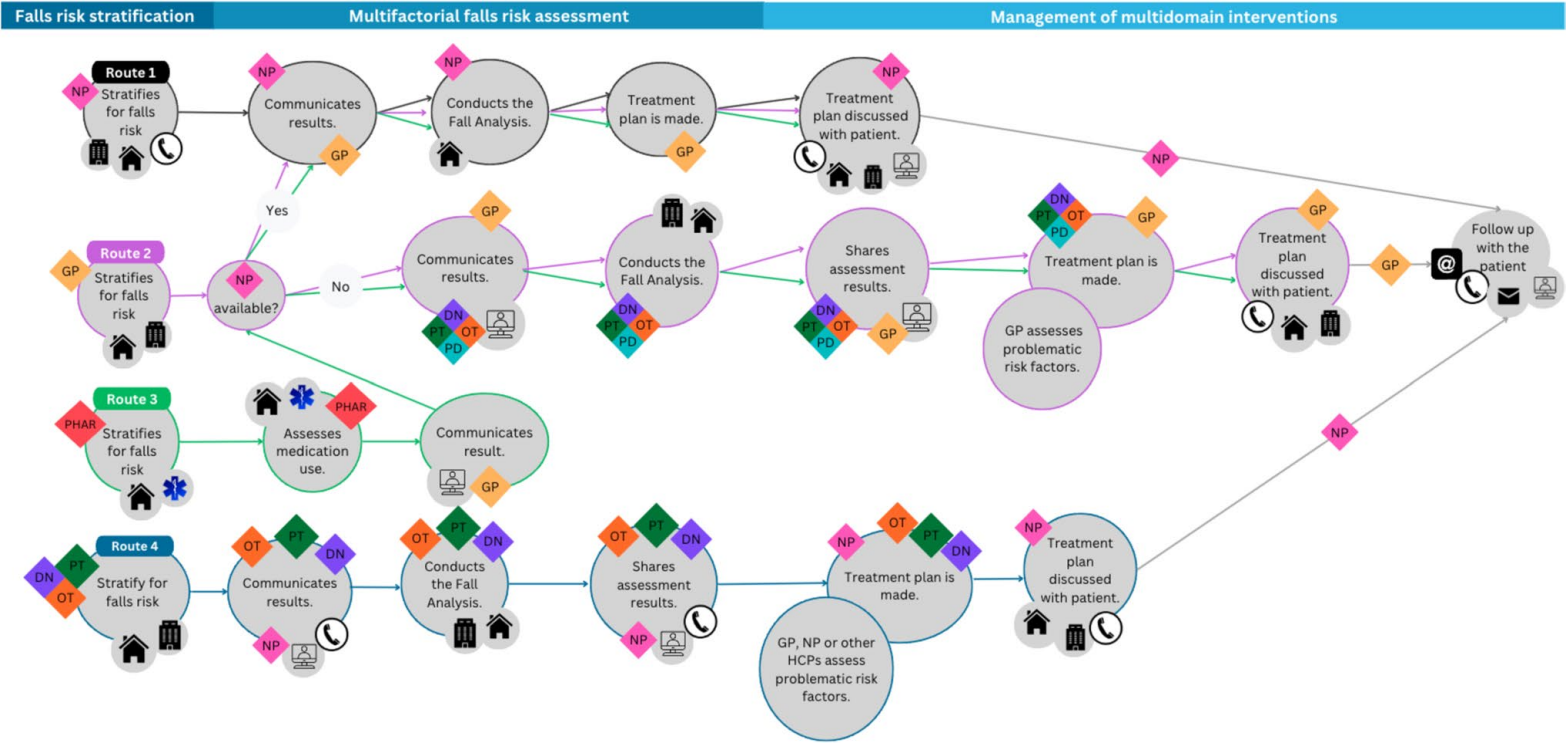
Journey map portraying the desired future state of multidisciplinary care pathways for falls prevention in primary care. Developed using Canva graphic design platform. *GP* general practitioner, *NP* nurse practitioner, *PHAR* pharmacist, *DN* district nurse, *PT* physical therapist, *OT* occupational therapist, *PD* podiatrist

Figure 1 illustrates the journey map portraying the desired future state of multidisciplinary care pathways for falls prevention that are in accordance with the needs of HCPs. A more detailed version of Fig. 1 can be found in the online appendix as Fig. S2. Table S1 in the online appendix displays the multidisciplinary falls prevention care needs of nurse practitioners, physical therapists, occupational therapists, podiatrists, pharmacists, district nurses, and the GP linked to each pathway component (i.e., falls risk stratification, multifactorial falls risk assessment, and management of multidomain interventions).

### Falls risk stratification: case manager

HCPs discussed having a role in falls risk stratification to identify high risk of falling in older adults (see routes 1–4). Pharmacists mentioned how they can stratify for falls risk when older adults pick-up their medications (see route 3). In turn, occupational therapists described how the social domain (e.g., informal caregivers, neighbors) sometimes initiated contact with them to conduct falls risk stratification in community-dwelling older adults (see route 4):

“And in any case, we have organized it in such a way that if, for example, an informal caregiver or a neighbour or the municipality is concerned about a particular client, they can simply contact us if the client also gives permission for the process to continue. […] So it’s not just that only HCPs can do it, because I think that actually, if you think of those three questions [in the falls risk stratification] and answer them, a lot of people can do it.”

Consensus was reached on the important role that GPs and nurse practitioners play in falls risk stratification in older adults, assigning the role of subsequent case manager to the nurse practitioner. This was in line with nurse practitioners who saw themselves as having the role of case manager for the patient, and had a preference for falls risk stratification via telephone due to efficiency (see route 1). The GP also viewed GP practices as a *“very good entry point for when an increased risk of falling is suspected”* as they *“know what”* falls prevention-related care *“[has] already been done”* by looking into the patient’s file to *“prevent things from being done twice.”*

### Multifactorial falls risk assessment: preparatory information and team size

HCPs discussed the usefulness of receiving preparatory patient information prior to the multifactorial falls risk assessment. Important preparatory information included *“entire medication list”* [physical therapist], *“medical history”* [occupational therapist], and/or previous administration of a cognition test. There was a preference for receiving preparatory information from the GP, which was also perceived as feasible by the GP as they can *“make a referral letter”* with *“the patient’s medical history included.”* However, an up-to-date list of medications should be requested *“from the pharmacy, not the GP”* [GP]. Additionally, HCPs mentioned the importance of a small multidisciplinary care team, consisting of two-to-three HCPs from different disciplines to reduce the burden on the patient. They also discussed how the assessment of certain risk factors should be carried out at the patient’s home, such as activities of daily living, resulting in one to two appointments per patient. Nurse practitioners preferred close collaboration with GPs and vice versa (see routes 1–2). According to the GP, nurse practitioners are *“the most logical and best person[s]”* for conducting a multifactorial falls risk assessment as *“they have access to the medical records”* and *“they work closely with the GP.”* However, in the event that the GP practice did not have access to a nurse practitioner, then collaboration should be sought with occupational therapists, physical therapists, district nurses, and/or podiatrists (see route 2). Occupational therapists, physical therapists, district nurses, and pharmacists perceived GPs, nurse practitioners and/or other HCPs (e.g., dieticians) as preferred collaborators for the assessment of risk factors that lie outside of their expertise, such as cognition, medication, dizziness/vestibular, mobility, nutritional status, and vitamin D intake (see routes 3–4). Specifically, the involvement of *“another professional or several colleagues”* would allow them to *“make decisions”* which would be *“helpful”* as they *“don’t have the expertise in everything”* [district nurse]. One pharmacist also explains:

“Basically, we [pharmacists] are the medication experts, and that is, of course, our profession. The physical therapist, on the other hand, can train muscles or work on people’s walking techniques, and an occupational therapist can assess a home where there are too many rugs lying around. So everyone should continue doing their thing; you just need to ensure that you collaborate with each other. […] You know, so you want that whole process around it to have a shared goal, so to speak. That’s where I see additional value: In doing falls prevention but each with their own specialization.”

### Management of multidomain interventions: GP and patient involvement

All HCPs mentioned that the involvement of a GP is standard for the management of multidomain interventions. For example, physical therapists, pharmacists, and nurse practitioners stated that they share the final report of the multifactorial falls risk assessment and recommendations for interventions tailored to detected risk factors with the GP. HCPs also discussed the importance of *“work[ing] together with the patient”* when *“decid[ing] which factors to address and when”* [GP]. According to the GP, follow-up should occur in 6 weeks post assessment by the nurse practitioner (i.e., the case manager of the patient). However, if an older patient needs one or more referrals to a specialist, this should always be made in accordance with the GP:

"Well, if the nurse practitioner thinks that a referral to a physical therapist is necessary, they will arrange that themselves. If they think there might be something for the hospital, then the patient comes to see me first in the consultation; I’ll listen to their heart or do a neurological examination, and then we look at what is already known. We discuss whether it makes sense to do a referral, and if so, whether it should be to a geriatrician, neurologist, or cardiologist—or maybe even to internal medicine?"

### Similarities and differences: beliefs, communication, and workload

Similarities and differences in HCP needs were observed when discussing the desired future state of multidisciplinary care pathways for falls prevention. Physical therapists and nurse practitioners emphasized how motivating HCPs to conduct annual falls risk stratification in a standardized manner is difficult, as such assessments are not considered standard practice. Moreover, there was also a belief among physical therapists that not every HCP has the qualifications to conduct a multifactorial falls risk assessment in older adults. According to many HCPs, good communication between HCPs is lacking in practice. Pharmacists discussed that they and other HCPs are not informed when *“someone has an increased risk of falling”* [pharmacist]. Similarly, district nurses indicated that sometimes they are not made aware by the GP of important changes to a patient’s file, such as a change in medications. While good communication facilitates the referral process for some nurse practitioners, others are unable to keep short lines of communication, such as not being notified that “*the physical therapist is on vacation for three weeks”* [nurse practitioner]. There was a need among many HCPs to integrate existing communication mediums, such as an online communication or referral platform that enables accessible collaboration between HCPs, informal caregivers and the patient, in multidisciplinary care pathways for falls prevention.

A difference between HCPs was the amount of risk factors in the multifactorial falls risk assessment and workload. District nurses expressed how collaboration between HCPs could help reduce the administration time of such an assessment in older adults. Similarly, according to the GP, being part of multidisciplinary falls prevention care is more feasible than conducting a multifactorial falls risk assessment alone arguing that *“the chances of GPs doing [the assessment] themselves are not very high”* due to *“extra work”* [GP]. By contrast, physical therapists were uncertain about the extent to which GPs should be included in the multidisciplinary care pathways for falls prevention due to workload. Finally, while nurse practitioners are open to the role of case manager, they also emphasized that *“there’s already plenty of work”* and feared that additional responsibilities, such as being part of multidisciplinary falls prevention care implies *“that even more work will come our way”* [nurse practitioner].

## Discussion

In this study, we sought to uncover primary care-based HCPs needs for multidisciplinary falls prevention care. Subsequently, these needs were used to develop a visual journey map outlining the desired future state of multidisciplinary care pathways for falls prevention by HCPs (i.e., for falls risk stratification, multifactorial falls risk assessment, and management of multidomain interventions). We found that a case manager program after risk stratification, preparatory patient information before the assessment, small multidisciplinary care team for the assessment, patient involvement during intervention management, good communication between HCPs, and a reduction in workload are essential for sustainable multidisciplinary care pathways for falls prevention. Research trials show that multidisciplinary care pathways for falls prevention for older adults are an effective falls prevention strategy [7–9, 23, 24], but their configuration in actual practice is suboptimal [11, 12, 25]. Using journey mapping, we were able to explore the optimum configuration of multidisciplinary care pathways for falls prevention for different HCPs, providing valuable insight for policy-makers seeking to improve these pathways in primary care. In this study, the need of a case manager for high-risk older adult fallers and increased patient involvement were found to be important components for improving multidisciplinary falls prevention care for all participating HCPs. Previous research has shown that a case management program can reduce falls in older adults through increased patient involvement [26]. Moreover, research has highlighted the facilitating role of patient involvement on adherence to multidomain falls prevention interventions in older adults [27]. Therefore, policy-makers should consider implementing a case manager program into multidisciplinary care pathways for falls prevention to facilitate patient involvement and intervention adherence in older adults. Based on the findings from this study, the role of case manager could be fulfilled by nurse practitioners.

Additionally, while we found that almost all participating HCPs saw a primary role for themselves in falls risk stratification in older adults, this was not the case for the multifactorial falls risk assessment in older adults. HCPs in this study preferred collaborating on the assessment with a small multidisciplinary care team (such as a GP with a physical therapist) or distributing the entire assessment to other HCPs due to reasons related to efficiency. Specifically, some HCPs believed that belonging to a multidisciplinary falls prevention care team could help decrease the workload due to the distribution of the assessment of falls risk factors, while others voiced that involvement in such a team could result in additional responsibilities and, consequently, increase workload. This finding showcases how, on one hand, collaboration can facilitate multifactorial falls risk assessment in older adults and, on the other hand, act as an inhibiting factor. HCPs mentioned how the efficiency of multifactorial falls risk assessment collaboration between HCPs could be improved through the receival of preparatory information from the GP about the older patient (e.g., medical history) prior to the assessment. Moreover, evidence suggests that inadequate collaboration in falls prevention can result due to poor communication between HCPs [11, 12, 25, 28, 29]. Corroborating previous findings, in this study, poor communication between HCPs often resulted in (1) unawareness of high falls risk patients or changes to treatment plans; (2) not knowing who is responsible for the assessment of which falls risk factor(s); and (3) an inefficient referral process for the treatment of detected falls risk factors. Thus, policy-makers seeking to improve collaboration in multidisciplinary falls prevention care pathways should consider providing HCPs with the necessary resources to enable good communication between HCPs. For example, workshops [30] or connecting multidisciplinary care pathways to existing communication platforms, as mentioned by HCPs in this study, have been shown to facilitate the implementation of falls prevention interventions [28, 30].

A strength of this study is the use of an innovative method to explore the optimum configuration of multidisciplinary care pathways for falls prevention in older adults for different HCPs. As a result, we were able to identify requirements for making these pathways sustainable in primary care. However, given that this study focused on multidisciplinary care pathways for falls prevention in primary care, we did not examine the multidisciplinary falls prevention care needs of secondary care-based HCPs (e.g., geriatricians). Additionally, despite occupational therapists emphasizing the important role that informal caregivers play in falls risk screening, a further limitation of this study is that we did not examine the multidisciplinary care pathways for falls prevention in the social domain (i.e., neighbors, municipalities, and informal caregivers). Future research should examine and visualize the requirements for sustainable pathways in the secondary care and social domain through journey mapping to improve the implementation of falls prevention interventions further.

Another strength of this study is the inclusion of different primary care-based HCPs. This allowed us to effectively capture the diverse needs of HCPs for multidisciplinary care pathways for falls prevention. However, the following limitations should be noted with regards to the sample of this study. First, among the primary care-based HCPs who participated in this study, only one was a practicing GP. Despite HCPs in this study highlighting the influential role of nurse practitioners as case managers, which is reflective of falls prevention in Dutch GP practices whereby certain tasks are distributed to nurse practitioners, the lack of participating GPs presents limitations to the generalizability of the findings. While the influential role of GPs for falls risk and factors management is well established across the literature [31], future research should explore the multidisciplinary falls prevention care needs of GPs. Second, despite nationwide sampling, information about the total number of HCPs who received the call for participants is unknown due to the privacy regulations of organizations. Therefore, selection bias cannot be ruled out, which presents limitations to the generalizability of the within-group findings. Specifically, HCPs who chose to participate in this study may be more motivated in falls prevention initiatives compared to those who did not participate. While motivation is recognized as a facilitator for the implementation of multidisciplinary care pathways for falls prevention [28], future research should examine and visualize the needs of less-motivated HCPs to enhance the implementation of falls prevention interventions further.

## Conclusion

This study developed a visual journey map outlining the desired future state of multidisciplinary care pathways for falls prevention in older adults by primary care-based HCPs (i.e., for falls risk stratification, multifactorial falls risk assessment, and management of multidomain interventions), providing important implications for policy-makers seeking to develop sustainable pathways in practice. Implementing a case manager program into multidisciplinary care pathways for falls prevention and providing HCPs with access to resources to enable good communication between HCPs, such as through workshops or connections to existing communication platforms, are fruitful for improving collaboration between HCPs.

## Supplementary Information

Below is the link to the electronic supplementary material.

Supplementary file1 (DOCX 497 KB)

## Data Availability

The data generated and analyzed during this study are not publicly available to ensure compliance with ethical guidelines and participant consent agreements. Requests for access to summarized data may be considered on a case-by-case basis, subject to appropriate ethical approval and fully executed data use agreements.
