# Supplementary material for: Multidisciplinary care pathways for falls prevention in older adults: visualizing the needs of primary care-based health care professionals
Source: Eur Geriatr Med. 2025 Jan 9;16(1):229–36. doi: 10.1007/s41999-024-01142-3 (PMC11850515; doi:10.1007/s41999-024-01142-3)
Supplement: Supplementary file 1 — Supplementary file1 (DOCX 497 KB) [file 41999_2024_1142_MOESM1_ESM.docx]

**Full list of organizations used for recruitment of HCPs**

| **Dutch names** | **English translations** |
| --- | --- |
| - VeiligheidNL | - Dutch Consumer Safety Institute |
| - Koninklijk Nederlands Genootschap voor Fysiotherapie (KNGF) | - Royal Dutch Society for Physical Therapy |
| - Landelijke Huisartsen Vereniging (LHV) | - National General Practitioners Association |
| - Ergotherapie Nederland | - Occupational Therapy Netherlands |
| - Nederlandse Vereniging voor Geriatrie Fysiotherapie (NVFG) | - Dutch Association for Geriatric Physical Therapy |
| - Verpleegkundigen en Verzorgenden Nederland (V&VN) | - Nurses and Care Assistants Netherlands |
| - Nederlandse Vereniging van Praktijkondersteuners en Praktijkverpleegkundigen (NVvPO) | - Dutch Association of Nurse Practitioners and Practice Assistants |
| - Koninklijke Nederlandse Maatschappij ter bevordering der Pharmacie (KNMP) | - Royal Dutch Society for the Advancement of Pharmacy |
| - Het Nederlands Huisartsen Genootschap (NHG) | - The Dutch General Practitioners Association |

**Semi-structured focus group/interview guide**

**Introduction**

- Who are you? (name, profession, region)
- What is your experience with screening and assessing falls risk and factors in older adults in practice?
- Tell me about a positive or negative experience you have had while screening and assessing falls risk and factors in older adults?
- Tell me why you decided to participate in this focus group?

**The Fall Analysis trajectory**

- Which health care professional group do you think is/are suitable for screening for falls risk in older adults?
  - Is it suitable to screen for falls risk via a telephone consultation? Why/why not? What are possible alternatives?
- Do you think that collecting and preparing relevant information about the patient related to the Fall Analysis would be helpful for you? Why/why not?
  - What preparatory information about the patient do you need to perform the Fall Analysis as optimally as possible? Consider the 13 risk factors that can be assessed using the Fall Analysis.
  - Through which means of communication would you like to receive/share preparatory information from/with other health care professionals?
- How would you like to conduct the Fall Analysis?
  - Should the Fall Analysis be performed in two appointments?
  - Where do the appointments take place, in the practice or at the client’s home?
  - Which location(s) is/are most suitable for assessing certain risk factors?
- Which health care professional group do you think is/are suitable for completing the Fall Analysis trajectory? Consider how the advice based on the results of the Fall Analysis should be communicated to the patient.
- To what extent does the Fall Analysis trajectory differ from your current trajectory for high falls risk?
- Which risk factors in the Fall Analysis are already part of the multifactorial falls risk assessment for older adults that you do in practice?
- Do you foresee problems with assessing certain risk factors in the Fall Analysis? For example, with the risk factors that you are less familiar with?
  - What are these problems? And, to which risk factor(s) does/do this apply?
  - What would help you to assess this/these risk factor(s)?

**Fig. S1** Journey map used for focus group and interviews


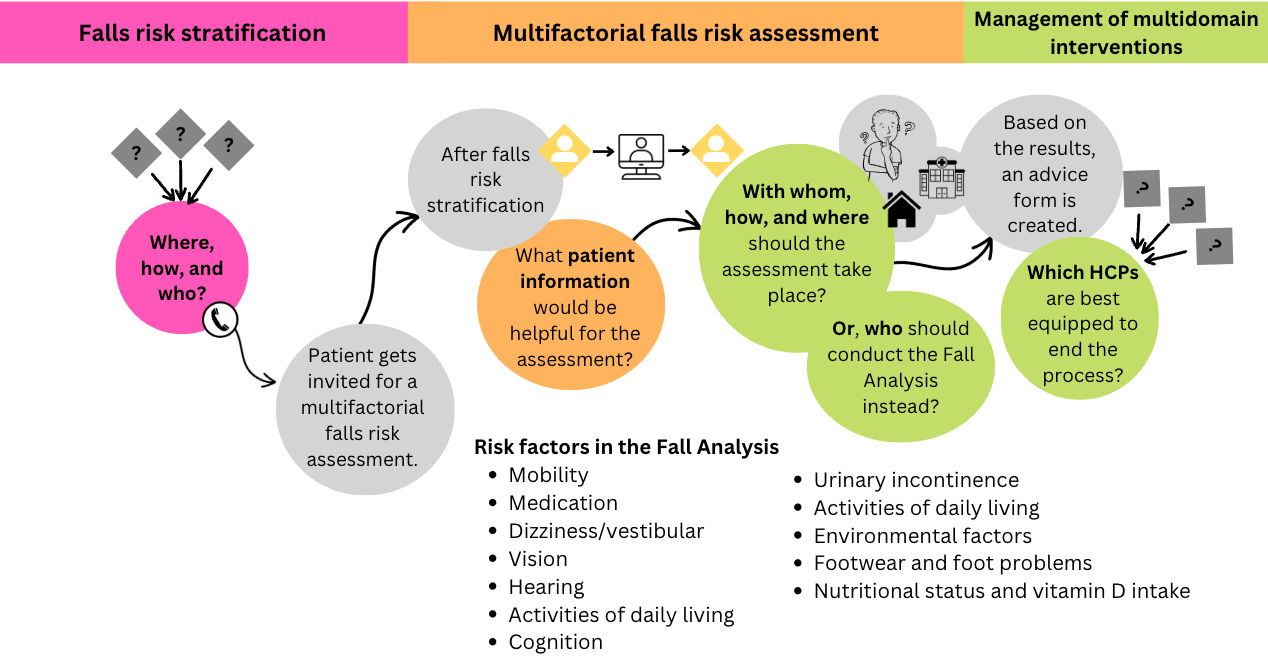


*Note*. Developed using Canva graphic design platform.

**Fig. S2** Detailed journey map portraying the desired future state of multidisciplinary care pathways for falls prevention in primary care


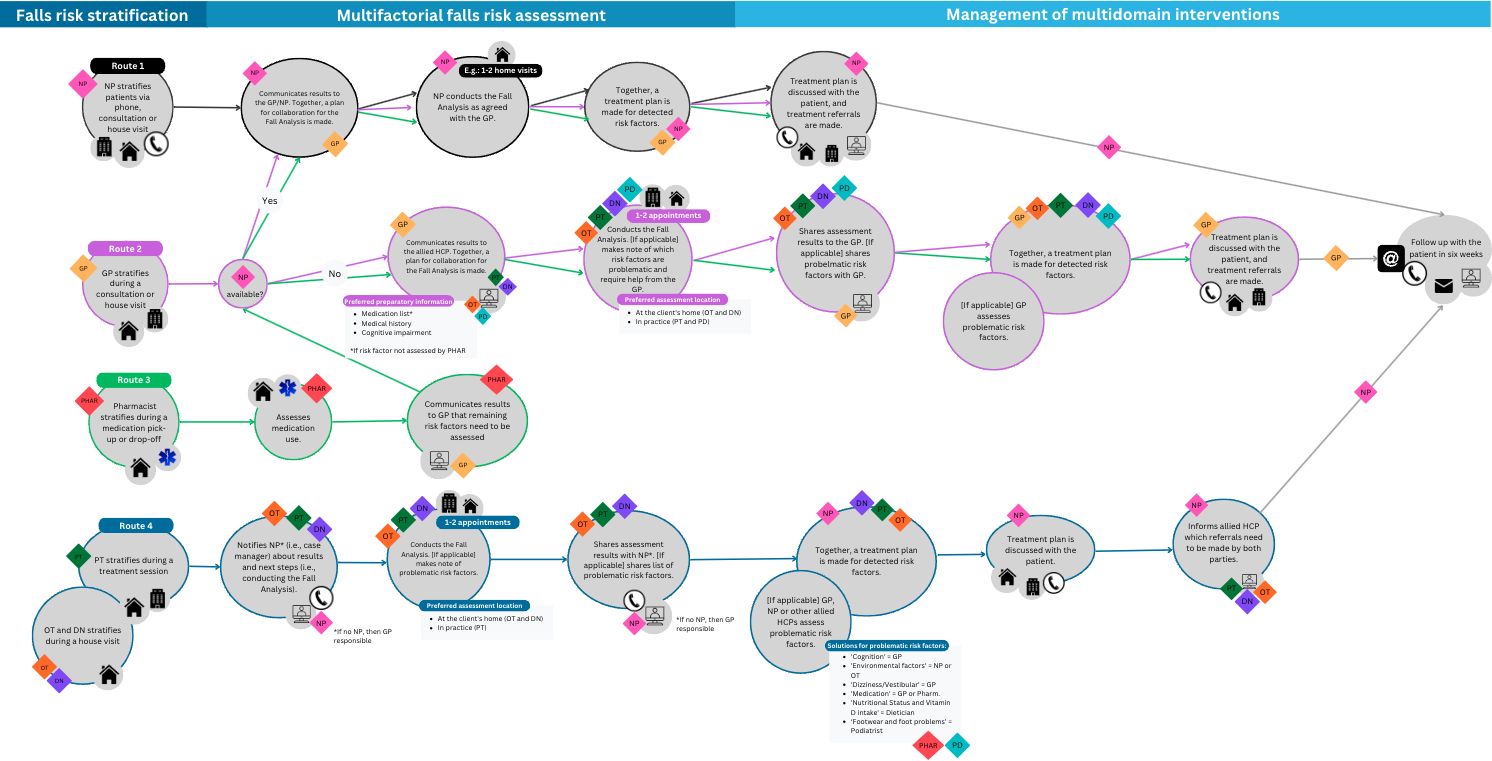


*Note*. Developed using Canva graphic design platform. Abbreviations: GP = general practitioner, NP = nurse practitioner, PHAR = pharmacist, DN = district nurse, PT = physical therapist, OT = occupational therapist, PD = podiatrist.

**Table S1** Multidisciplinary falls prevention care needs of HCPs

| **Topic** | **HCP quote** |
| --- | --- |
| Falls risk stratification | *“Additionally, when stratifying for falls risk, I think the big advantage of a pharmacy is of course that a lot, we have a lot of contact moments with the patients at risk. Whether that is a [medication] review or an extra evaluation or a first prescription or a second prescription, or manual sales, a lot of high-risk patients come to the pharmacy at least once a year. So, if you want to capture that entire group, a pharmacy would be a logical starting point. I think.”*  – Pharmacist |
|  | *“I think, you actually want a HCP who is responsible for the entire Fall Analysis both for guidance and afterward. A great example within podiatry is diabetic foot care. […] The initial stratification is usually done by the* ***nurse practitioner****, and if they notice an increased risk of wounds, it is then referred to the podiatrist. At that point, the podiatrist becomes the case manager for the diabetic foot care of that patient. This means the patient comes to us, we conduct all possible tests to assess the risk of developing wounds, and then we can determine what care the patient needs to avoid wounds. […] It could work similarly here, not necessarily for podiatrists but for all other HCPs. You screen for falls risk, and if it's identified that someone may have an increased risk, then you have a specific HCP who becomes the case manager for the Fall Analysis and its guidance.”*  – Podiatrist |
|  | *“[…] I think that the GP and the* ***nurse practitioner*** *need to be tackled the most. To create that awareness when patients actually come there for blood pressure or something, that the risk of falling is also discussed.”*  – Physical therapist |
|  | *“But I do think that you are a HCP group that is primarily responsible for ensuring that everything with your patients is going well. At least, I think that is my job as a* ***nurse practitioner*** *to follow up [with my older patients].”*  – **Nurse practitioner** |
|  | *“As a* ***nurse*** ***practitioner*** *for older adults, […] I call the older adults over 75. I just do that in alphabetical order and I then ask them the three questions.”*  – **Nurse practitioner** |
|  | *“Yes, I do think that the GP practice is a very good entry point for when an increased risk of falling is suspected, also because we know what has already been done. When I see that a patient has fallen then I look into the file and I think: ‘Oh, that [falls preventive care] was already completely mapped out by the* ***nurse practitioner*** *two years ago.’ So you also want to prevent things from being done twice and most of the [patient’s] history and such is known at the GP practice.”*  - GP |
| Preparatory patient information | *“From the GP, I always receive the entire medication list, so I don't ask for it any further, if it is not available I ask the patient. And then I work it out further at home to see how many medications are involved. Is there polypharmacy and which falls risk medications are present? And, I indeed mention that in my conclusion. And, my advice then adds: ‘Please monitor medication. Preferably once a year.’”*  – Physical therapist |
|  | *“[…] Of course you have to look at information, such as medication use and so on, incontinence. Yes, these are things that are often known to the GP.”*  – Podiatrist |
|  | *“In principle we get this [patient information] through the GP. […] A history in that sense is of course important, whether there has been an operation or something. Or whether there have already been other medical issues. So I always think a medical history is very important.”*  – Occupational therapist |
|  | *“And if, as a GP, you would need to make some kind of referral letter, that could work. You could make a referral letter just like when you refer someone to, I don't know, a specialist. You create a referral letter, and ideally, the GP would refer via [name of referral platform], which is a referral platform linked to our patient records. So I can just log into [name of referral platform] directly from a patient's file, and it's done automatically. Then I can also select which information should be included in the letter. That way, you immediately have the patient's medical history included.”*  - GP |
|  | *“Well, in any case, it’s important to know the medical history. Are there any heart problems, neurological issues, mobility problems, and cognition? Additionally, there's the medication, but you should request that from the pharmacy, not the GP. That’s also what we always did…; [name of* ***nurse practitioner****] would always request the medication from the pharmacy, because patients often have no idea what they are taking. And what’s also important, but you might get that from your conversation during the Fall Analysis, is understanding how someone functions in daily life. Also, is their daily life limited by any falls risk? So, to what extent is someone frail or not.”*  - GP |
| Multifactorial falls risk assessment | *“We actually make a home visit quite quickly to see how, apart from mobility, what the home situation looks like and to see whether there are already safety rails, stair lifts, alarms available and whether there is a need for them in consultation with the patient.”*  – **Nurse practitioner** |
|  | *“And that also makes it very easy and clear for us to map activities of daily living and environmental factors because you actually see the client at home in his own environment […]. So I think that it is a prerequisite that you do the Fall Analysis in the client's home situation.”*  – Occupational therapist |
| Size of multidisciplinary care team | *“Yes, yes, I would say it would be better to have some overlap than to miss something and that means people don't have to go to 10 different HCPs just to get all those questions or the whole Fall Analysis completed.”*  – Occupational therapist |
|  | *“[…] I would indeed not keep it with more than two or three people. But then I still think it is also very important to make some kind of plan with the patient and then look back. Okay, so what is most needed? What is compensated?”*  – Podiatrist |
|  | *“And if older patients indicate, "Oh yes, I would like to do something about that [my falls risk]," then I will discuss it with the GP, and that's where further analysis on various fronts [would begin]. […] People are categorized into high, low risk groups, etc. and then a whole process is set in motion.”*  – **Nurse practitioner** |
|  | *“Yes, you know, in principle, I think the* ***nurse practitioner*** *is the most logical and best person for this. Because they have the insight, they have access to the medical records, they work closely with the GP, and they are right there in primary care.”*  - GP |
|  | *“And then the alternative solutions are more due to a capacity problem... If the Netherlands considers this [multifactorial falls risk assessment] so important and the capacity for* ***nurse practitioners*** *is limited, then the financing should also be reviewed. Or perhaps some of the tasks that currently fall to the* ***nurse practitioner*** *should be shifted to district nurses, for example.”*  - GP |
|  | *“I think cognition is the biggest problem, because I find it confrontational to assess. So that is a point: Should we assess it, when are you going to assess it? You need the Montreal Cognitive Assessment or the Mini Mental State Exam for that. But I always find that very difficult for myself. […]So if I have any doubts, I'll go to the GP.”*  – Physical therapist |
|  | *“And I would rather go to a GP than to a pharmacist because the GP is much more able to see whether medication can be reduced or can be switched to a different type of medication.”*  – District nurse |
|  | *“All I do about dizziness is ask about it. And if necessary, ask the* ***nurse practitioner*** *or the GP to check orthostatic hypotension.”*  – Occupational therapist |
|  | *“Nutrition also goes to the dietician, for me.”*  – Physical therapist |
|  | *“I almost never do those mobility tests. I find that very difficult. […]And I think it doesn't quite feel like I should do them, I would rather have a physical therapist do them.”*  – Occupational therapist |
|  | *“For example, as a podiatrist, I can already address seven things. Then I can look, ‘Oh, who can this person turn to for the last five things? Oh, wait, I have a good connection with that person,’ so I see it more as not assigning specific tasks to each individual, but more like, ‘Oh, this person can help with these five things with that other person.’ I see it more as an ‘and, and’ story.”*  – Podiatrist |
|  | *“I work a lot with* ***nurse practitioners*** *from the GP’s office, and then they handle a portion of the assessment while I handle another portion.”*  – Physical therapist |
|  | *“I think it will help me make decisions... so indeed, because I don’t have the expertise in everything, I think this will be helpful. […]Yes, it [a multidisciplinary care approach] also allows me to involve a colleague right away, like saying, ‘well, it’s multidisciplinary,’ so it also involves another professional or several colleagues.”*  - District nurse |
|  | *“Yes, we are of course very much concerned with medication-related falls risk. But if you read the falls risk from VeiligheidNL then it, of course, has many other aspects, and for us this now means that it is picked up by the physical therapist or* ***nurse practitioner****.”*  – Pharmacist |
| Management of multidomain interventions | *“Yes, I type it into my file. So, I include the falls risk factors there, type them into my file, and then copy and paste them into my email to the GP.”*  – Physical therapist |
|  | *“And, of course, after your [medication] review, you always consult with the GP. So, in the end, I think the GP still has the lead because they can assess which issue we're addressing now, for example, falls risk, are we inviting people further, and what are we going to discuss with that person. These are the points that arise from the discussion between the pharmacist and the GP. So, on the one hand, I think maybe as a pharmacist, wanting to be involved throughout the entire project might be too much, but we also need to take this opportunity to showcase our role here and not let it pass us by.”*  – Pharmacist |
|  | *“And then a whole process is set in motion, and one of the components is that the physical therapist is linked to it in the context of falls training, so to speak. That's one part, but polypharmacy is another part. So we have multiple components, but then it's actually out of my hands at that moment after I've discussed it with the GP.”*  – **Nurse practitioner** |
|  | *“Yes, if you don't have contact with the other disciplines, then I think the GP should handle it, but you can discuss it with the patient beforehand, ensuring they are comfortable with it, that it will happen. Otherwise, you're just administering the Fall Analysis - 'okay, thank you, thank you... you'll hear from the GP’ - I personally don't find that a pleasant way of working.”*  – Physical therapist |
|  | *“What we used to do when we still had the multidisciplinary meeting was to hold the meeting, where each discipline would provide their recommendations. The* ***nurse practitioner*** *would then collect those recommendations, talk to the patient about what was discussed, and ask, 'What would you like to focus on, and what do you want to tackle first?' So, essentially prioritizing with the patient. Most of the time, things like sleeping pills and alcohol are low on the priority list, so we might say, 'Let’s first focus on something like training with the physical therapist,' and then follow up with a call after six weeks, asking, 'So, how’s it going? Is it getting better? Maybe now it’s time to consider reducing the sleeping pills?' Something like that. So, you work together with the patient to decide which factors to address and when.”*  - GP |
| Similarities and differences  Beliefs | *“What [name] is saying is that the implementation of behavior change takes a lot of time. It's about raising awareness among everyone about how important this [falls risk stratification] is and looking at what is needed to make this standard practice.”*  - Physical therapist |
|  | *“Yeah, I always find it difficult when things get standardized or something because it's so individual where you are in that situation. I'm also a bit allergic to it when I have to fill out tests myself. Because you get this kind of vague idea of what you're supposed to do, and you think: 'Well, that’s just completely useless to me.”*  - **Nurse practitioner** |
|  | *“[…] What I'm a bit afraid of is that certain professionals, a physical therapist or occupational therapist or anyone else who is not very skilled with it, will conduct a multifactorial falls risk assessment in older adults. I think you have to have some experience with it.”*  - Physical therapist |
| Communication between HCPs | *“I think a lot of problems also arise from the lack of data sharing in primary care. […] once you agree that someone has an increased risk of falling, it would of course be nice if every HCP knew that”*  - Pharmacist |
|  | *“…you might learn certain things because clients tell you…when you visit a client and you're not involved in their medication, there could easily be changes in their medication without you knowing. Because a GP doesn't always inform us, the client has to do that themselves. So there are definitely outcomes that we don't know about.”*  - District nurse |
|  | *“Fortunately in our neighborhood, contact with the occupational therapist is very accessible, so that is just very nice. If in doubt, we can contact an occupational therapist very quickly within a few weeks or within two weeks.”*  - **Nurse practitioner** |
|  | *“But also, you know, to ensure that the plans you set up or that have been set up are being followed. And if the physical therapist is on vacation for three weeks, that you know about it, so that something else can be arranged, or that you can communicate that back. … So a lot of things are left undone, and that's really unfortunate.”*  - **Nurse practitioner** |
|  | *“I work for a GP and also a lot with [name of communication platform] and I think that this is a nice tool to use for someone who has fallen and where you then work on the problem with several professionals, so to speak. And, you also get good information, can share, and it is transparent for the patient and informal caregiver.”*  - District nurse |
|  | *"But it would be great if it's a document that can be filled in by professionals, and also by multiple professionals from different disciplines. That way, you can make a request to the GP, like, 'Okay, I need to complete this multifactorial falls risk assessment, could you fill in this and that information?'"*  - Occupational therapist |
|  | *“I definitely think it [reducing the administration time of the Fall Analysis] can help within a network. I work together with physical therapists, district nurses, and the elderly care network, and I think part of it falls under GP care—I work for the GPs—and then you can divide it up nicely. […] I think you can look closely at your network to see who can handle what. […] I think a multidisciplinary approach can really work well. Then we have everything...and as a check, we can see who can best address specific issues for that older adult at the moment.”*  - District nurse |
| Workload | *“I think the chances of GPs doing it themselves are not very high because it’s definitely extra work. How long it takes also depends on the patient, as it often involves a broad range of issues. It's not just a matter of quickly ticking off questions; there's usually a whole story around it. But in principle, if someone has an increased falls risk, having the GP practice as the first point of contact is a good idea, I think.”*  **-** GP |
|  | *“What gives me a bit of a strange feeling is the involvement of the GP because from practice we know that GPs are so incredibly overloaded. I really wonder if that [GPs as case managers] is the ideal option for the future.”*  - Physical therapist |
|  | *“Yes, there's already plenty of work, so if you start encouraging more [multifactorial falls risk assessment for older adults], I always worry that even more work will come our way.”*  - **Nurse practitioner** |
